# Influence of Early Postoperative Basal Insulin Treatment and Post-Transplant Diabetes Mellitus Risk on Health-Related Quality of Life in Kidney Transplant Recipients—An Analysis of Data From a Randomized Controlled Trial

**DOI:** 10.3389/ti.2023.11370

**Published:** 2023-08-03

**Authors:** Balazs Odler, Matthias Huemer, Elisabeth Schwaiger, Andrea Borenich, Amelie Kurnikowski, Marcell Krall, Hildegard Hafner-Giessauf, Georgios Eleftheriadis, Friderike Bachmann, Anna Faura, María José Pérez-Sáez, Julio Pascual, Klemens Budde, Alexander R. Rosenkranz, Manfred Hecking, Kathrin Eller

**Affiliations:** ^1^ Division of Nephrology, Department of Internal Medicine, Medical University of Graz, Graz, Austria; ^2^ Palliative Care Unit Associated With the Division of Oncology, Department of Internal Medicine, Medical University of Graz, Graz, Austria; ^3^ Department of Internal Medicine III, Clinical Division of Nephrology and Dialysis, Medical University of Vienna, Vienna, Austria; ^4^ Department of Internal Medicine II, Kepler University Hospital, Med Campus III, Linz, Austria; ^5^ Institute for Medical Informatics, Statistics and Documentation, Medical University of Graz, Graz, Austria; ^6^ Department of Nephrology and Medical Intensive Care, Charité Universitätsmedizin Berlin, Berlin, Germany; ^7^ Department of Nephrology, Hospital del Mar, Institute Mar for Medical Research, Barcelona, Spain

**Keywords:** kidney transplantation, HRQOL, insulin, PTDM, clinical study

## Abstract

Health-related quality of life (HRQOL) improves after kidney transplantation (KT) but declines over time. Studies on the effect of early postoperative basal insulin therapy on HRQOL after KT, especially KTRs at high risk of developing post-transplant diabetes mellitus (PTDM) are missing. Data from a randomized controlled trial on 148 non-diabetic KTRs were analyzed. HRQOL using the KDQOL-SF™ was compared in KTRs who either received early postoperative basal insulin therapy or standard-of-care and in KTRs at risk of developing PTDM. Determinants of HRQOL outcomes were investigated using multivariable linear regression analysis. In total, 148 patients completed the KDQOL-SF at baseline. Standard-of-care or early basal insulin therapy after KT did not influence HRQOL. Overall, KT improved the mental (MCS) and physical component summary (PCS) scores at 6-month after KT, which remained stable during further follow-up visits. However, patients at high-risk for PTDM had significantly greater impairment in the PCS score (baseline, 24 months) without differences in MCS scores. In the multivariable regression analysis, allograft function and hemoglobin levels were associated with decreased MCS and PCS scores, respectively. A limitation of the study is the fact that only around 50% of the ITP-NODAT study patients participated in the HRQOL evaluation. Still, our data clearly show that early basal insulin therapy does not affect HRQOL after KT but is negatively influenced by classical clinical factors and PTDM-risk at 24 months after KT. The latter might be influenced by older age.

## Graphical Abstract

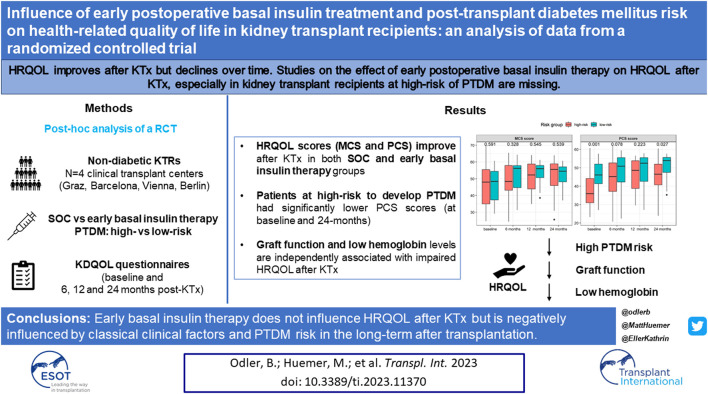

## Introduction

Chronic kidney disease (CKD) has a major impact on both physical and mental health, especially in patients with advanced CKD including dialysis dependency [1–4]. Both reduced self-reported and objective physical function as well as mental health are associated with increased mortality rates in this patient population [5]. Kidney transplantation (KT) is considered the optimal and most cost-effective treatment in patients with advanced CKD with improved survival rates and clear benefits on quality of life (QOL) measures [6, 7].

Although clinically relevant improvements are substantive, a proportion of KTRs experience poor health-related quality of life (HRQOL) despite ongoing satisfactory allograft function [8, 9]. In addition, recent evidence suggests a decline in patients’ HRQOL in the long-term after KT [10]. Some clinical and psychological factors occurring in a substantial proportion of KTRs such as airflow limitation [11], gastrointestinal symptoms [12], side effects of immunosuppressive drugs [13], or anxiety [9] are well-documented to negatively affect HRQOL.

Post-transplant diabetes mellitus (PTDM) is a frequent complication associated with mortality in individuals after KT [14–16]. While the impact of PTDM on graft loss is debated [17], clear associations on cardiovascular (CV) outcomes, especially in patients with modifiable CV risk factors such as obesity are well known [18–21]. The appropriate management of PTDM remains challenging and has previously been reviewed [22, 23]. Neutral Protamin Hagedorn (NPH) insulin and insulin analogs to treat hyperglycemia are commonly used in the early post-transplant period, however, these require intensive blood glucose monitoring and patients’ adherence to avoid therapy-associated adverse events [24, 25].

According to the American Diabetes Association (ADA), HRQOL is a key measure, which should be integrated into the care of patients with diabetes mellitus (DM) in order to improve management and clinical outcomes [26]. Although the occurrence and challenges on treatment of PTDM in KTRs are well recognized, little is known about the impact of this complication on HRQOL of patients after KT. In line, many uncertainties on interventions improving HRQOL of KTRs remain unresolved. Thus, assessing modifiable risk factors and interventions to improve general health status and prevent decline in HRQOL are highly desired.

In this study, we aimed to investigate whether the application of early postoperative basal insulin therapy for the prevention of PTDM might affect HRQOL compared with standard care in KTR using long-term, protocoled HRQOL data obtained in the Insulin Therapy for the Prevention of New Onset Diabetes after Transplantation (ITP-NODAT) study [27]. Moreover, we evaluated the contributors of HRQOL in individuals at high-risk for developing PTDM after KT. Since certain KTRs might benefit from tailored basal insulin therapy, such data are important to allow for more individualized recommendations regarding PTDM prophylaxis strategies after KT.

## Methods

### Study Design

The detailed description of the original study protocol (ClinicalTrials.gov registration: NCT03507829) and the primary results have been published previously [27]. Briefly, the ITP-NODAT study was an investigator initiated, open label, prospective, randomized, multi-center clinical trial with an unblinded end-point evaluation to test the efficacy of early postoperative basal insulin therapy for the prevention of PTDM in KTRs. Four clinical transplant centers (Medical University of Vienna, Austria; Medical University of Graz, Austria; Hospital del Mar Barcelona, Spain; Charité Universitätsmedizin Berlin, Germany) participated in the study. Patients were randomized in a 1:1 ratio in each participating center prior to transplantation and stratified by first versus repeated kidney transplant. The 24-month follow-up was finalized in May 2020 and the primary results were published in 2021 [27].

### Participants and Interventions

Detailed patient eligibility and the study interventions have been described previously [27]. In brief, *n* = 263 non-diabetic KTRs receiving standard immunosuppressive therapy (tacrolimus, mycophenolate, and steroids) were included in the study. After randomization, patients were divided into standard of care control and treatment groups. In the standard of care control group, once daily fasting plasma glucose monitoring was performed, and antihyperglycemic treatment was initiated according to the physician`s decision. In contrast, KTRs in the treatment group underwent regular capillary blood glucose monitoring (4-times daily) and received basal insulin therapy with intermediate acting (NPH) insulin (human insulin isophane, Humulin N [Eli Lilly]) combined with short-acting insulin (insulin lispro, Humalog [Eli Lilly]), if the afternoon (pre-supper) glucose values exceeded 140 mg/dL (7.8 mmol/L). Pre-specified dose adjustment schemes for insulin titration and application of antihyperglycemic medication for both study groups, as well as predefined schemes for immunosuppression, were applied in each participating center as described previously [27]. Predefined trial visits were performed at 3, 6, 12, and 24 months after KT.

### Study Definitions

PTDM was defined as 2 h post oral glucose tolerance test ≥200 mg/dL (11.1 mmol/L) or hemoglobin A1c (HbA1c) ≥6.5% (48 mmol/mol) according to the ADA guideline criteria [26]. Patients at high risk of developing PTDM after KT were defined using age, serum lipid levels, body mass index (BMI), family history of DM, and the history of polycystic kidney disease (PCKD) based on previously published literature data [27]. Accordingly, patients fulfilling at least one of the following criteria at the time of transplantation were defined as part of the high-risk population:

1. Age ≥ 60 years
2. Age 45–59 plus one of the following criteria: triglycerides ≥200 mg/dL *or* triglycerides 150–200 mg/dL and BMI>27 *or* triglycerides 150–200 mg/dL and high-density lipoprotein (HDL) < 40 mg/dL (men) *or* triglycerides 150–200 mg/dL and HDL<50 mg/dL (women)
3. Family History of DM
4. Polycystic kidney disease (PCKD)

### Assessment

HRQOL was evaluated using the kidney disease quality of life short form (KDQOL-SF™) [28]. The KDQOL-SF is a multidimensional patient reported outcome measurement. It is available in different languages including Spanish and German. The questionnaire was developed to assess the health-related disease burden of individuals with CKD and on dialysis with excellent psychometric properties (Cronbach’s alpha = 0.61–0.90). The multiple scales of the questionnaire include 43 disease-targeted items focusing on symptoms, effects of kidney disease, burden of kidney disease, work status, cognitive function, quality of social interaction, sexual function, sleep, social support, dialysis staff encouragement, and patient satisfaction. Additionally, the questionnaire includes the short form 36-health survey (SF-36™). The scoring of each scale ranges from zero to 100 and can be calculated if at least 50% of each scales’ items were completed by the participant. Higher scores reflect better self-reported QOL.

The KDQOL-SF measurements were self-reported and assessed at baseline, and during the trial visits at 6, 12, and 24-month follow-up. All assessments were carried out in parallel to collection of trial data in the parent trial at the respective study site.

### Outcomes

In the original study, pre-specified primary and secondary endpoints were defined at month 12 and 24 post-transplant, respectively and were published previously [27]. The HRQOL was defined as secondary endpoint in the original study as the SF-36 mental component summary (MCS) and SF-36 physical component summary (PCS) scores derived from the KDQOL-SF at 6, 12, and 24 months after KT [27]. Exploratory outcomes include change in the KDQOL-SF subscales in the predefined study groups in the parent trial and in the low- and high-risk groups for PTDM at the same follow-up time points.

### Statistical Analysis

Patient characteristics were reported as absolute and relative frequencies for categorical data and for numerical data as means and standard deviation (SD) if normally distributed or medians (range) otherwise. Comparison between groups were done using t-tests, Mann-Whitney U tests, Chi-square, Wilcoxon signed-rank tests or Fisher’s exact tests as appropriate. Univariable and multivariable linear regression analyses were performed for physical and mental component scores at 6, 12, and 24 months after kidney transplantation. Treatment group, baseline PCS and MCS scores, risk group (for PTDM), renal function (eGFR), hemoglobin, inflammation (CRP), and glycemic control (HbA1c, OGTT) were included in the univariable analysis as covariates. All variables with a *p*-value <0.2 in the univariable analysis were included in the multivariable analysis. Beta coefficients were presented along with their 95% confidence interval (CI). A *p*-value of 0.05 or less was considered statistically significant. All statistical analyses were conducted using R version 4.2.

## Results

### Patient Characteristics in the Study Groups Stratified by Standard-of-Care and Treatment

The total study sample involved 73 and 75 participants (*N* = 148 in total, 56.3% overall response rate) in the standard of care and treatment groups, respectively. The low response rate resulted from patient incompliance, if they were unable to fill out the questionnaires or incomplete questionnaires. Baseline characteristics of the participants were balanced between the groups randomized to standard of care control or treatment groups. Participants in the treatment group had a higher proportion of polycystic kidney disease (PCKD) as primary kidney disease and tended to have a higher rate of DM in the family history, and had lower body weight as well as BMI. Patients’ characteristics at baseline in the whole cohort and study groups are summarized in Table 1.

**TABLE 1:** Baseline patient characteristics in the standard of care and treatment groups.

Characteristic	Overall *N* = 148	Control *N* = 73	Treatment *N* = 75	*p*-value
Female	55 (37)	25 (34)	30 (40)	0.5
Age (years)	49.9 (13.9)	50.4 (14.5)	49.4 (13.3)	0.6
Height (cm)	169 (10)	169 (10)	170 (10)	0.6
Weight (kg)	71.2 (63.5, 82.0)	76.4 (66.0, 83.5)	68.4 (63.0, 77.0)	**0.017**
BMI (kg/m^2^)	25.5 (4.6)	26.5 (5.2)	24.6 (3.7)	**0.019**
Primary kidney disease				0.054
Glomerular	59 (56)	33 (66)	26 (47)	
Vascular	11 (10)	5 (10)	6 (11)	
Tubulointerstitial	9 (9)	5 (10)	4 (7)	
PCKD	23 (22)	5 (10)	18 (33)	
Other	3 (3)	2 (4)	1 (2)	
Number of previous kidney allografts				0.3
1	126 (85)	60 (82)	66 (88)	
2	20 (14)	12 (16)	8 (11)	
3	1 (1)	0 (0)	1 (1)	
4	1 (1)	1 (1)	0 (0)	
Dialysis prior to KT	134 (91)	63 (86)	71 (95)	0.082
Comorbidities				
Cardiovascular	60 (41)	31 (42)	29 (39)	0.6
Respiratory	11 (7)	7 (10)	4 (5)	0.3
Urinary	14 (10)	8 (11)	6 (8)	0.5
Endocrinological	18 (12)	7 (10)	11 (15)	0.3
Neurological	3 (2)	1 (1)	2 (3)	>0.9
Psychiatrical	8 (5)	1 (1)	7 (9)	0.063
Other	6 (4)	4 (6)	2 (3)	0.4
Laboratory results				
Hemoglobin (g/dL)	11.9 (1.5)	11.6 (1.5)	12.1 (1.5)	0.12
Creatinine (mg/dL)	7.4 (5.8, 9.5)	7.1 (5.4, 9.2)	7.7 (6.1, 9.5)	0.2
eGFR (ml/min/1.73m^2^)	7.2 (5.3, 9.3)	7.6 (5.6, 10.1)	6.6 (4.8, 8.8)	0.3
CRP (mg/dL)	2.0 (0.6, 6.7)	1.6 (0.6, 5.8)	2.0 (0.8, 6.8)	0.5
HbA1c (%)	5.2 (4.8, 5.4)	5.3 (4.8, 5.5)	5.1 (4.9, 5.3)	0.4

Statistically significant *p*-values in the analysis appear in bold (*p* < 0.05). Continuous variables are expressed as mean (SD) or median (minimum and maximum). Categorical variables are n (%).

Abbreviations: BMI, body mass index; CRP, C-Reactive Protein; eGFR, estimated glomerular filtration rate; HbA1c, hemoglobin A1c; KT, kidney transplantation; PCKD, polycystic kidney disease.

### HRQOL Measures in the Study Groups Stratified by Standard of Care and Treatment

**FIGURE 1:**
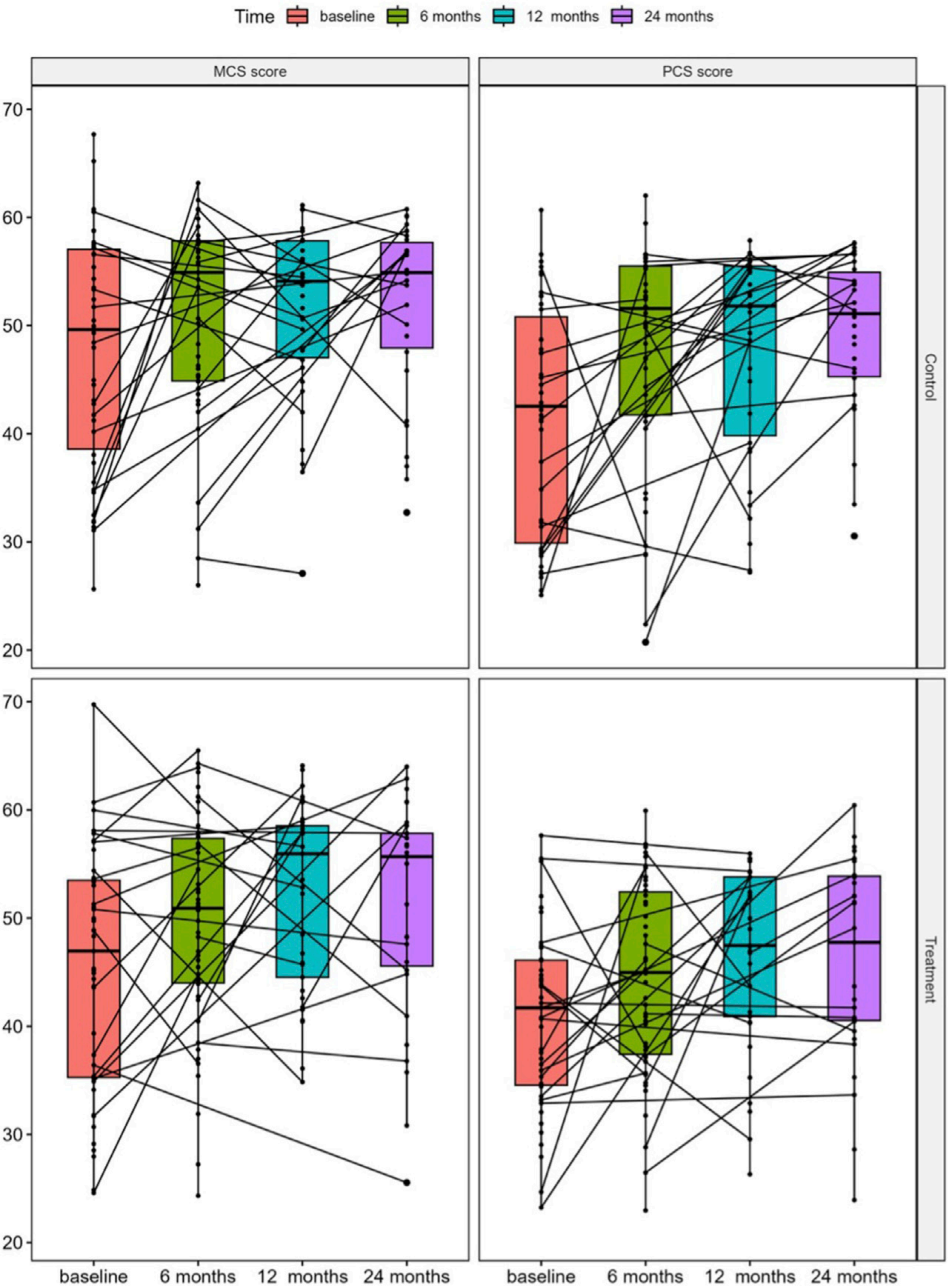
Median changes in KDQOL-SF SF-36 physical component summary (PCS) and mental component summary (MCS) scores in the study groups stratified by standard of care control and treatment at baseline and at 6, 12, and 24 months after KT.

PCS and MCS scores were calculated from available and valid responses for 85 (57%) participants at baseline, 91 (61%) at 6-month, 67 (45%) at 12-month and 61 (41%) at 24-month follow-up. Missing data resulted mainly from patients lost to follow-up or incomplete questionnaires. In both, the standard of care control and the treatment groups, a significant increase in MCS [control: 54.9 (26.0, 63.2) vs. 49.6 (25.6, 67.7), *p* = 0.046 and treatment: 50.9 (24.3, 65.5) vs. 46.9 (24.6, 69.7), *p* = 0.004, respectively] and PCS [control: 51.6 (20.7, 62.0) vs. 42.5 (25.1, 60.7), *p* = 0.001 and treatment: 45.0 (23.0, 59.9) vs. 41.7 (23.2, 57.6) *p* = 0.015, respectively] scores were observed at 6-month as compared to baseline, which remained stable during the further follow-up visits (Figure 1).

### Baseline Patient Characteristics in the Low-, and High-Risk Groups

Within the study sample, 65% of the patients were defined as high-risk for developing PTDM. Detailed characteristics of the resampled study sample concerning the risk factors are provided in Table 2. The high-risk group included predominantly males (60%, *n* = 50), had a mean age of 56.4 years (SD: 12.5 years), 41% had glomerular disease as a primary kidney disease and had higher baseline HbA1c. Baseline patient characteristics were similar between high- and low-risk groups (Table 2).

**TABLE 2:** Baseline patient characteristics in the groups of low- and high-risk for post-transplant diabetes mellitus.

Characteristic	High-risk *N* = 84	Low-risk *N* = 45	*p*-value
Female	34 (40)	16 (36)	0.6
Age (years)	56.4 (12.5)	39.7 (10.4)	**<0.001**
Height (cm)	168.4 (10.3)	170.9 (10.4)	0.2
Weight (kg)	75.0 (65.3, 84.7)	70.0 (62.0, 79.0)	0.062
BMI (kg/m^2^)	26.6 (4.8)	23.8 (3.8)	**0.002**
Primary kidney disease			**<0.001**
Glomerular	26 (41)	22 (71)	
Vascular	7 (11)	4 (13)	
Tubulointerstitial	5 (8)	4 (13)	
PCKD	23 (37)	0 (0)	
Other	2 (3)	1 (3)	
Number of previous kidney allografts			0.9
1	71 (85)	40 (89)	
2	12 (14)	5 (11)	
3	0 (0)	0 (0)	
4	1 (1)	0 (0)	
Dialysis prior to KT	75 (89)	40 (89)	>0.9
Comorbidities			
Cardiovascular	38 (45)	15 (33)	0.2
Respiratory	9 (11)	2 (4)	0.3
Urinary	9 (11)	4 (9)	>0.9
Endocrinological	13 (15)	3 (7)	0.15
Neurological	2 (2)	1 (2)	>0.9
Psychiatrical	7 (8)	0 (0)	0.10
Other	2 (2)	3 (7)	0.3
Laboratory results			
Hemoglobin (g/dL)	11.7 (1.5)	12.0 (1.6)	0.2
Creatinine (mg/dL)	7.1 (5.6, 9.3)	7.8 (6.2, 9.7)	0.3
eGFR (ml/min/1.73m^2^)	7.1 (5.1, 9.0)	7.2 (5.5, 9.3)	0.6
CRP (mg/dL)	2.0 (0.6, 10.0)	1.7 (0.6, 3.4)	0.3
HbA1c (%)	5.3 (5.0, 5.5)	4.8 (4.6, 5.2)	**<0.001**

Statistically significant *p*-values in the analysis appear in bold (*p* < 0.05). Continuous variables are expressed as mean (SD) or median (minimum and maximum). Categorical variables are n (%).

Abbreviations: BMI, body mass index; CRP, C-Reactive Protein; eGFR, estimated glomerular filtration rate; HbA1c, hemoglobin A1c; KT, kidney transplantation; PCKD, polycystic kidney disease.

### HRQOL Measures in the Groups of High- and Low-Risk for PTDM

**FIGURE 2:**
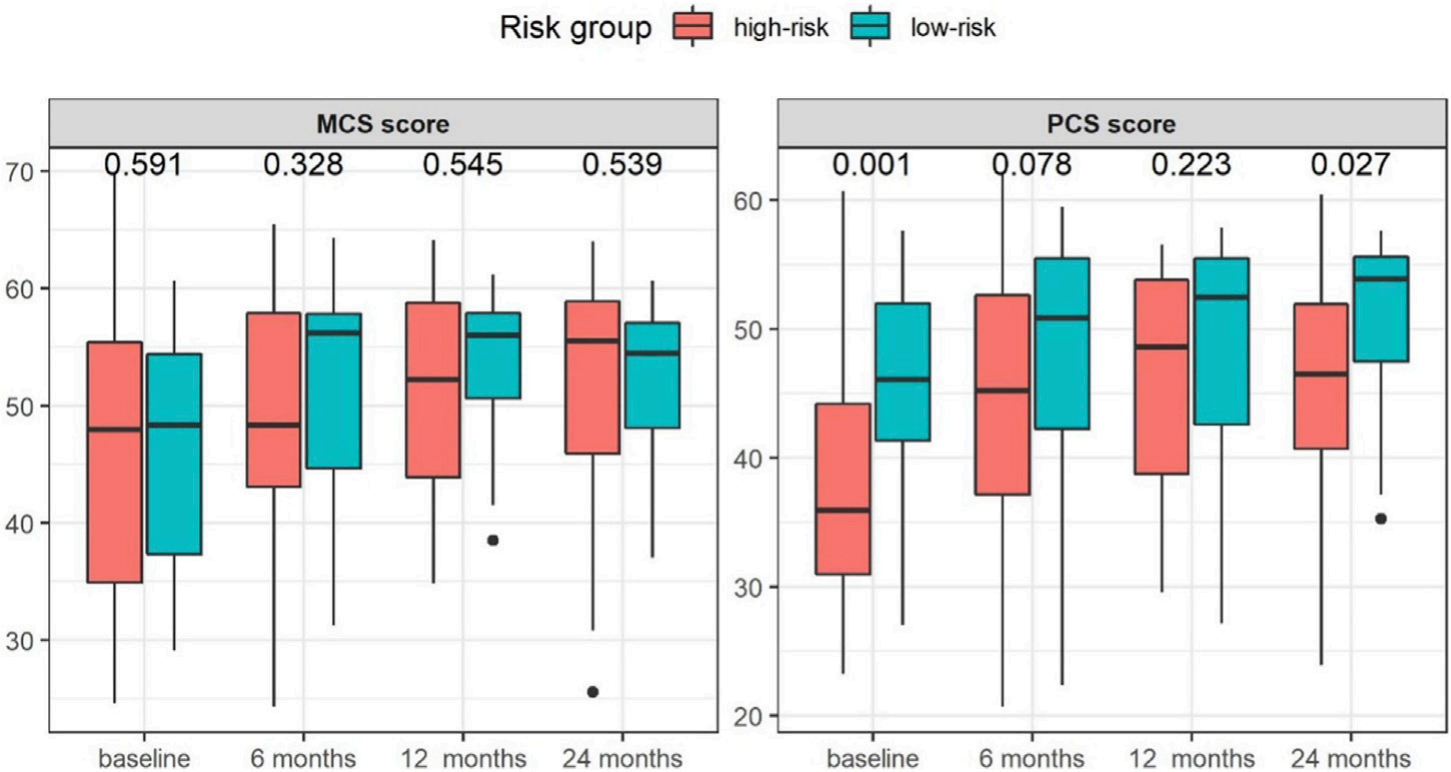
Median changes in KDQOL-SF SF-36 physical component summary (PCS) and mental component summary (MCS) scores in the study groups stratified by risk group for post-transplant diabetes mellitus at baseline and at 6, 12, and 24 months after KT.

Patients in the high-risk group had a significantly greater impairment in the PCS scores at baseline [35.9 (23.2, 60.7) vs. 46.1 (27.1, 57.6), *p* < 0.001] and 24 months after transplantation [46.5 (23.9, 60.4) vs. 53.9 (35.3, 57.7), *p* = 0.027] as shown in Figure 2. No significant differences in the MCS scores [baseline: 48.0 (24.6, 69.7) vs. 48.3 (29.1, 60.7), *p* = 0.591 and 24 months: 55.5 (25.5, 64.0) vs. 54.5 (37.0, 60.7), *p* = 0.539] were found.

**FIGURE 3:**
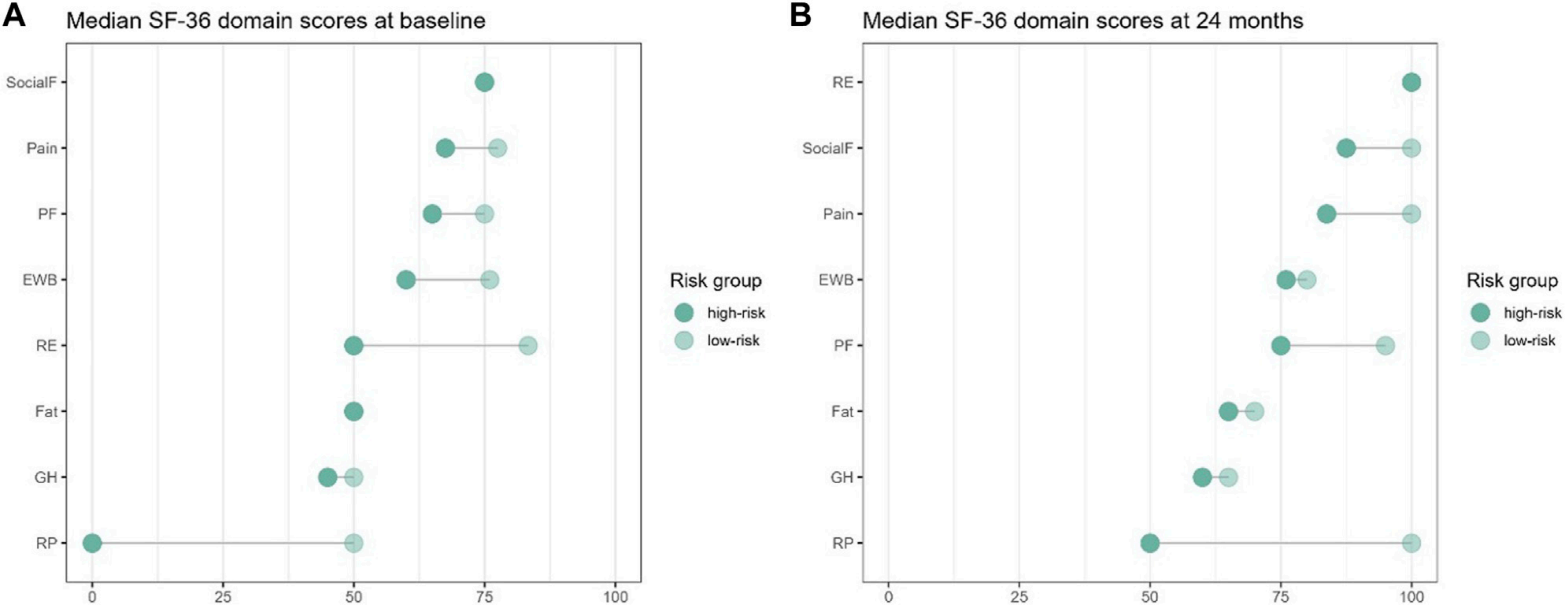
Changes of median KDQOL-SF SF-36 domain scores at **(A)** baseline (BL) and **(B)** 24-month for low-risk compared to high-risk for post-transplant diabetes mellitus.

The PCS and MCS scores were comparable between high- and low-risk groups at baseline (Figure 3A), only showing a significant difference in the score of physical-role-functioning [0.0 (0.0, 100.0) vs. 50.0 (0.0, 100.0), *p* = 0.001] at this time-point. At 24 months after transplantation, significant differences in pain [77.5 (20.0, 100.0) vs. 100.0 (32.5, 100.0), *p* = 0.045], physical-role-functioning [50.0 (0.0, 100.0) vs. 100.0 (0.0, 100.0), *p* = 0.003], and emotional-role-functioning [100.0 (0.0, 100.0) vs. 100.0 (0.0, 100.0), *p* = 0.030] between the high-risk and the low-risk groups were found (Figure 3B).

Within the disease specific scores of the KDQOL-SF™, there were no significant differences between the scores of the symptom problem list [84.1 (43.2, 100.0) vs. 95.5 (56.8, 100.0), *p* = 0.064], effects of kidney disease [87.5 (53.1, 100.0) vs. 87.5 (37.5, 100.0), *p* = 0.453], burden of kidney disease [81.2 (6.2, 100.0) vs. 84.4 (25.0, 100.0), *p* = 0.946], work status [50.0 (0.0, 100.0) vs. 100.0 (0.0, 100.0), *p* = 0.076], cognitive function [86.7 (26.7, 100.0) vs. 93.3 (46.7, 100.0), *p* = 0.671], quality of social interaction [86.7 (46.7, 100.0) vs. 86.7 (53.3, 100.0), *p* = 0.586], sexual function [62.5 (0.0, 100.0) vs. 100.0 (12.5, 100.0), *p* = 0.236], sleep [63.8 (32.5, 100.0) vs. 74.2 (25.0, 92.5), *p* = 0.110], and overall health [80.0 (30.0, 100.0) vs. 80.0 (50.0, 100.0), *p* = 0.196] at 24 months.

### Confounders of HRQOL Measures

In the univariable regression analysis, early postoperative insulin treatment was not significantly associated with the PCS or MCS scores at any timepoint but being in the low-risk group for developing PTDM was significantly related to a better PCS score at 24 months after transplantation (Beta: 5.1, 95% CI: 0.54–9.6, *p* = 0.029). Hemoglobin, renal function, CRP, baseline PCS and MCS scores, and OGTT were significantly associated with PCS and MCS scores at various timepoints (Table 3).

**TABLE 3:** Univariable regression analyses of confounders for changes in KDQOL-SF™ physical and mental component scores at 6, 12, and 24 months after kidney transplantation.

	Physical component summary score								
	Univariable								
	*N*	m6 Beta, (95% CI)	*p*-value	*N*	m12 Beta, (95% CI)	*p*-value	*N*	m24 Beta, (95% CI)	*p*-value
Group	91		0.120	67		0.541	61		0.136
Control		—			—			—	
Treatment		−3.2 (−7.2, 0.84)			−1.4 (−5.8, 3.1)			−3.2 (−7.4, 1.0)	
Risk group	81		0.128	61		0.320	52		**0.029**
High-risk		—			—			—	
Low-risk		3.6 (−1.1, 8.2)			2.3 (−2.3, 7.0)			5.1 (0.54, 9.6)	
Baseline PCS	48	0.42 (0.13, 0.71)	**0.006**	43	0.24 (−0.04, 0.52)	0.095	40	0.35 (0.05, 0.66)	**0.025**
HbA1c	87	0.61 (−2.3, 3.5)	0.677	62	−1.5 (−5.1, 2.0)	0.384	58	−2.6 (−6.5, 1.4)	0.194
eGFR	89	0.02 (−0.05, 0.09)	0.599	67	0.18 (0.09, 0.28)	**<0.001**	61	0.18 (0.09, 0.28)	**<0.001**
Hemoglobin	86	1.6 (−0.54, 2.7)	**0.004**	64	−1.2 (−0.02, 2.4)	**0.046**	59	2.0 (0.72, 3.2)	**0.003**
CRP	85	−0.1 (−0.18, −0.01)	**0.024**	67	−0.29 (−0.82, 0.25)	0.291	61	−0.26 (−0.54, 0.02)	0.067
oGTT	91	0.02 (−0.01, 0.05)	0.232	67	−0.02 (−0.05, 0.02)	0.314	61	0.00 (−0.08, 0.08)	0.973

	Mental Component Summary Score								
	Univariable								
	*N*	m6 Beta, (95% CI)	*p*-value	*N*	m12 Beta, (95% CI)	*p*-value	*N*	m24 Beta, (95% CI)	*p*-value
Group	91		0.507	67		0.775	61		0.797
Control		—			—			—	
Treatment		−1.4 (−5.4, 2.7)			0.59 (−3.5, 4.7)			−0.60 (−5.3, 4.1)	
Risk group	81		0.170	61		0.249	52		0.856
High-risk		—			—			—	
Low-risk		3.2 (−1.4, 7.9)			2.4 (−1.7, 6.6)			0.45 (−4.5, 5.4)	
Baseline MCS	48	0.41 (0.16, 0.67)	**0.002**	43	0.38 (0.16, 0.59)	**0.001**	40	0.18 (−0.08, 0.44)	0.178
HbA1c	87	−0.29 (−3.2, 2.6)	0.845	62	2.2 (−0.86, 5.2)	0.158	58	−0.85 (−4.9, 3.2)	0.677
eGFR	89	−0.03 (−0.04, 0.09)	0.445	67	0.08 (−0.02, 0.17)	0.107	61	0.12 (0.01, 0.23)	**0.034**
Hemoglobin	86	1.2 (0.11, 2.3)	**0.032**	64	0.91 (−0.22, 2.0)	0.111	59	0.59 (−0.88, 2.1)	0.427
CRP	85	−0.05 (−0.13, 0.04)	0.259	67	−0.01 (−0.51, 0.49)	0.968	61	−0.10 (−0.42, 0.21)	0.508
oGTT	91	−0.03 (−0.07, 0.00)	**0.042**	67	0.00 (−0.03, 0.04)	0.884	61	−0.01 (−0.10, 0.07)	0.781

Statistically significant *p*-values in the analysis appear in bold (*p* < 0.05). Only variables with a *p*-value <0.2 in the univariable analysis were carried on for multivariable analysis.

Numbers represent the beta coefficients and 95% confidence intervals in each group and variable, while *N* represents the number of patients with available data.

Abbreviations: BMI, body mass index; CRP, C-Reactive Protein; eGFR, estimated glomerular filtration rate; HbA1c, hemoglobin A1c; MCS, mental component score; PCKD, polycystic kidney disease; PCS, physical component score.

Neither the PCS nor the MCS scores were associated with early postoperative insulin treatment or risk-profile in our multivariable analysis (Table 4). However, our model showed a significant association of baseline PCS score at 6 months (Beta: 0.40, 95% CI: 0.04–0.76, *p* = 0.029) renal function at 12 months (Beta: 0.14, 95% CI: 0.02–0.27, *p* = 0.025), and hemoglobin at 24 months (Beta: 3.3, 95% CI: 1.50–5.10, *p* < 0.001) after transplantation with PCS score, while baseline MCS score (Beta: 0.32, 95% CI: 0.06–0.59, *p* = 0.017) and hemoglobin at 6 months (Beta: 2.6, 95% CI: 0.71–4.60, *p* = 0.009) as well as renal function at 24 months (Beta: 0.19, 95% CI: 0.05–0.32, *p* = 0.008) after KT were significantly associated with MCS score (Table 4).

**TABLE 4:** Multivariable regression analyses of confounders for changes in KDQOL-SF™ physical and mental component scores at 6, 12, and 24 months after kidney transplantation.

	Physical component summary score								
	Multivariable								
	*N*	m6 Beta, (95% CI)	*p*-value	*N*	m12 Beta, (95% CI)	*p*-value	*N*	m24 Beta, (95% CI)	*p*-value
Group	91		0.162	67			61		0.272
Control		—			—			—	
Treatment		−4.5 (−11.0, 1.9)						−2.6 (−7.4, 2.2)	
Risk group	81		0.856	61			52		0.604
High-risk		—			—			—	
Low-risk		−0.61 (−7.4, 6.2)			—	—		1.9 (−5.4, 9.1)	
Baseline PCS	48	0.40 (0.04, 0.76)	**0.029**	43	0.16 (−0.12, 0.44)	0.257	40	0.18 (−0.18, 0.54)	0.318
HbA1c	87	—		62	—	—	58	−4.0 (−9.0, 0.90)	0.105
eGFR	89	—		67	0.14 (0.02, 0.27)	**0.025**	61	0.06 (−0.07, 0.20)	0.360
Hemoglobin	86	1.6 (−0.55, 3.7)	0.139	64	0.31 (−1.2, 1.9)	0.688	59	3.3 (1.5, 5.1)	**<0.001**
CRP	85	0.07 (−0.15–0.29)	0.521	67	—	—	61	0.49 (−0.02, 1.0)	0.060
oGTT	91	—		67	—	—	61	—	—

	Mental Component Summary Score								
	Multivariable								
	*N*	m6 Beta, (95% CI)	*p*-value	*N*	m12 Beta, (95% CI)	*p*-value	*N*	m24 Beta, (95% CI)	*p*-value
Group	91		—	67		—	61		—
Control		—			—			—	
Treatment		—							
Risk group	81		0.160	61		—	52		—
High-risk		—			—			—	
Low-risk		4.2 (−1.7, 10)			—			—	
Baseline MCS	48	0.32 (0.06, 0.59)	**0.017**	43	0.21 (−0.07, 0.49)	0.142	40	0.07 (−0.19, 0.32)	0.592
HbA1c	87	—	—	62	0.41 (−3.6, 4.5)	0.836	58	—	—
eGFR	89	—	—	67	0.06 (−0.08, 0.20)	0.401	61	0.19 (0.05, 0.32)	**0.008**
Hemoglobin	86	2.6 (0.71, 4.6)	**0.009**	64	0.60 (−1.3, 2.5)	0.518	59	—	—
CRP	85	—	—	67	—	—	61	—	—
oGTT	91	−0.03 (−0.07, 0.01)	0.138	67	—	—	61	—	—

Statistically significant *p*-values in the analysis appear in bold (*p* < 0.05). Only variables with a *p*-value <0.2 in the univariable analysis were carried on for multivariable analysis.

Numbers represent the beta coefficients and 95% confidence intervals in each group and variable, while N represents the number of patients with available data.

Abbreviations: BMI, body mass index; CRP, C-Reactive Protein; eGFR, estimated glomerular filtration rate; HbA1c, hemoglobin A1c; MCS, mental component score; PCKD, polycystic kidney disease; PCS, physical component score.

## Discussion

In this analysis of data from a randomized controlled trial [27], the overall HRQOL of the study population remained stable during the follow-up period but increased significantly in the PCS after KT. In addition, KTRs at high risk of developing PTDM had a significantly greater impairment in physical functioning at baseline and 24 months after KT. Most importantly, early postoperative insulin therapy was not associated with worse HRQOL measures, but kidney allograft function and associated anemia were independent predictors of reduced PCS and MCS scores at certain time points during the 2 years follow-up after KT.

The risk of hypoglycemic complications in PTDM patients was found to be comparable to other types of DM [29], and basal insulin treatment can negatively affect HRQOL by inducing symptomatic albeit mild hypoglycemia [30]. In the ITP-NODAT trial [27], the postoperative basal insulin therapy was initiated early with a preventive intention requiring a stringent risk-benefit assessment. Early basal insulin treatment did show significantly higher hypoglycemic events in the treatment group, but mainly within the first 3 months of post-operative treatment. Some patients escalated their insulin dosage without clear indication resulting in seriously low blood sugar levels and suggesting an insecurity with the handling of insulin at the beginning of the treatment. However, with ongoing treatment no further hypoglycemic events were registered [27]. In accordance, the results of our analysis indicate no negative effect of early basal insulin therapy on the HRQOL of the patients. These data are of critical significance since we need a personalized glucose-lowering therapy for different patient cohorts according to their risk profile without negatively affecting HRQOL.

A possible explanation for why early basal insulin therapy does not affect HRQOL in KT recipients despite having similar diabetes and hypoglycemic associated complications may be a stronger positive effect of KT itself [29, 30]. Basal insulin therapy and the accompanied fear of hypoglycemic events is known to negatively affect the HRQOL of non-transplant diabetes patients. While these fears may also appear in KT recipients, the overall positive effects of transplantation including cessation of hemodialysis, increased physical functioning, and decreased effects and burden of kidney disease may outweigh the negative effects of early basal insulin therapy in post-operative KT patients [9].

To further explore the effects of early postoperative insulin therapy on HRQOL, we resampled the study participants according to their risk of developing PTDM using the factors age and clinical predisposition for metabolic dysfunction in addition to laboratory markers. Metabolic dysfunction may be the result of an unfavorable lifestyle or a genetic predisposition including a positive family history of DM. In line, PCKD as a systemic genetic disorder resulting in a progressive growth of cysts not only in the kidneys, but also in the liver, seminal ducts, and/or pancreas are at high-risk to develop PTDM [31, 32]. Nevertheless, metabolic dysfunction and overweight are considered as a distinct pathological entity, the metabolic syndrome, which itself affects the HRQOL of patients [33, 34]. The resulting two groups of high versus low-risk for the development of PTDM showed significant differences in their physical health (SF-36 PCS) 24 months after KT, while there were no differences in mental health (MCS) at any timepoint. Interestingly, at 6 and 12 months after transplantation, both groups showed increased and comparable HRQOL scores, suggesting a valuable benefit of KT during the first year for all patients. Positive changes in HRQOL observed early after KT are particularly driven by increased physical activity, reduced symptom burden or improvements in social functioning, among others [10]. In the long-term course after KT, a significant impairment of physical health, which is associated with a pronounced muscular weakness resulting in a lower physical activity has been—comparable to our results—proven before [9].

The cause of muscular weakness in KTR is multifactorial. Older patients experience geriatric syndromes like sarcopenia, which itself, has a complex pathophysiology including reduced physical activity, systemic inflammation, and neuropathic changes leading to a denervation of muscles [35]. However, in younger patients, the metabolic syndrome and obesity are known to induce a loss of muscular strength relative to their body mass, which is again linked to systemic inflammation and reduced physical activity [35, 36]. Pro-inflammatory cytokines such as tumor-necrosis factor alpha and interleukin 6, are known to stimulate muscle protein degradation and reduce muscle protein synthesis. Pro-inflammatory states have all been described in sarcopenia [37], metabolic syndrome, obesity [38], and PCKD [32], which define the high-risk PTDM group. Accordingly, CRP levels tended to increase in the high-risk group in both the uni- and multivariable model 24 months after KT. The resulting sarcopenia together with the pre-existing metabolic syndrome might potentially explain our findings of reduced physical health 24 months after KT in the high-risk PTDM group. A potential strategy to prevent muscle weakness, sarcopenia, and metabolic risk factors in KT recipients could be controlled physical exercise. Recent randomized controlled trials investigating the effect of a 10–12 weeks training program of either resistance or combined resistance and aerobic exercise compared to no training in post KT showed notable improvements in functional performance, body composition, muscular strength, renal function, fatigue, and HRQOL [39–41]. Promoting physical activity in KT recipients may therefore prevent the observed vanishing effect of KT in high-risk PTDM patients.

The high-risk group also had a greater disease specific symptom burden, pain, and impaired emotional-role-functioning 24 months after KT compared to the low-risk PTDM group, with comparable values between groups at 6 and 12 months, suggesting again a slowly vanishing effect of KT on HRQOL in the high-risk PTDM group. We also found a statistical association of renal function (eGFR) and hemoglobin levels to the HRQOL 24 months after KT independent from early insulin treatment and PTDM-risk in the multivariable analysis. Hemoglobin levels were associated with the physical health domain (PCS score), while renal function was related to the mental health domain (MCS score) of the SF-36. The symptom burden of KTRs influencing HRQOL is multifactorial including decreased renal function, anemia, depressive symptoms, anxiety, and elevated BMI, as well as treatment-specific encompassing side effects and complications of immunosuppressive therapy [42, 43]. Both severely impaired renal function and anemia lead to uremic symptoms, fatigue, and breathlessness [43, 44], while immunosuppressive agents and metabolic syndrome can cause peripheral neuropathy, thereby enhancing pain and reducing mobility [45]. Particularly calcineurin-inhibitors may induce a disabling pain-syndrome in 5%–15% of KTRs within the first year after KT [46]. Psychological symptoms constitute a burden to patients *per se* but also enhance the perception of physical symptoms in a multidimensional way [47], together potentially creating a vicious cycle amplifying the total symptom burden after KT. The high-risk PTDM group of our study cohort were older and displayed a higher BMI, both associated with more depressive symptoms [33]. Additionally, metabolic dysregulation decreases renal function of the graft in the long-term [48], causing fears of graft rejection and anxiety further enhancing depressive symptoms [49, 50].

Our study has certain limitations including a considerable proportion of missing data within the HRQOL questionnaires, especially towards the end of the study period which might lead to underestimation to detect lower effect sizes. This is mainly explained by patients lost to follow-up or incompliance with completing the questionnaires. Furthermore, this analysis lacks socioeconomic data as well as comparison of different ethnicities since Caucasians were in the majority included in the ITP-NODAT study [27]. Sociodemographic differences may especially affect the definition of metabolic risk profiles limiting our results mainly to Caucasians living in Europe. In addition, the measurement instrument is generic and might miss to capture important diabetes-specific aspects. Nevertheless, the strengths of this analysis are its multicenter randomized design comprising detailed clinical data and the long-term follow-up of the study participants providing unique data on determinants of HRQOL on the long-term after KT.

Taken together, early postoperative insulin therapy, which might be reasonable in selected patient groups, is not compromising the HRQOL. Although KT substantially improves the HRQOL of CKD patients 1 year after transplantation, patients in the high-risk PTDM group experience a significant impairment in HRQOL in the long course after KT. The HRQOL of KTR is significantly dependent on graft function and anemia. Given the complex relationship between manageable risk factors, physical and psychological symptom burden, and nephrological treatment, a multidisciplinary post-transplant care should be considered to meet the multidimensional needs of KTR, especially within high-risk PTDM populations.

## Data Availability

The raw data supporting the conclusion of this article will be made available by the authors, without undue reservation.
